# Mitochondrial alterations in the cochlea of *Cdk5rap1*‐knockout mice with age‐related hearing loss

**DOI:** 10.1002/2211-5463.13655

**Published:** 2023-06-06

**Authors:** Toru Miwa, Tatsuya Katsuno, Fan‐Yan Wei, Kazuhito Tomizawa

**Affiliations:** ^1^ Department of Otolaryngology‐Head and Neck Surgery, Graduate School of Medicine Kyoto University Japan; ^2^ Department of Otolaryngology‐Head and Neck Surgery Osaka Metropolitan University Japan; ^3^ Department of Molecular Physiology, Faculty of Life Sciences Kumamoto University Japan; ^4^ Department of Modomics Biology and Medicine, Institute of Development, Aging and Cancer Tohoku University Sendai Japan

**Keywords:** age‐related hearing loss, *Cdk5rap1*, microstructural findings, mitochondria, mitochondrial tRNA, transmission electron microscopy

## Abstract

Previous studies have revealed that age‐related hearing loss (AHL) in Cdk5 regulatory subunit‐associated protein 1 (*Cdk5rap1*)‐knockout mice is associated with pathology in the cochlea. Here, we aimed to identify mitochondrial alterations in the cochlea of *Cdk5rap1*‐knockout mice with AHL. Mitochondria in the spiral ganglion neurons (SGNs) and hair cells (HCs) were normal despite senescence; however, the mitochondria of types I, II, and IV spiral ligament fibrocytes were ballooned, damaged, and ballooned, respectively, in the stria vascularis. Our results suggest that the accumulation of dysfunctional mitochondria in the lateral wall, rather than the loss of HCs and SGNs, leads to the onset of AHL. Our results provide valuable information regarding the underlying mechanisms of AHL and the relationship between aberrant tRNA modification‐induced hearing loss and mitochondrial dysfunction.

## Graphical abstract

This research aimed to reveal the mitochondrial alterations in the cochlea of *Cdk5rap1*‐knockout mice with age‐related hearing loss (AHL). We found that the loss of spiral ganglion neurons and hair cells is not the cause of AHL. Rather, the accumulation of dysfunctional mitochondria in the lateral wall leads to the onset of AHL.

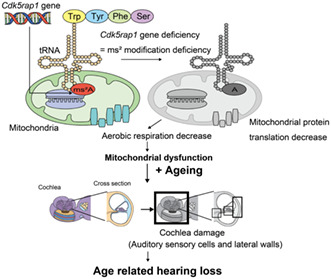

**Abbreviations**
AHL: age‐related hearing loss
CDK5RAP1: Cdk5 regulatory subunit‐associated protein 1
CNT: control
EP: endolymphatic potential
GLUT: glutaraldehyde
HC: hair cell
IHC: inner hair cell
KO: knockout
ms^2^
: 2‐methylthio
mt‐tRNA: mitochondrial tRNA
OHC: outer hair cell
SGN: spiral ganglion neuron
SLi: spiral ligament
SV: stria vascularis
TEM: transmission electron microscopy

Age‐related hearing loss (AHL) is a multifactorial condition. Some studies have indicated that altered mitochondrial DNA, imbalanced mitochondrial redox, reactive oxygen species production, and modified antioxidant capacity occur due to age‐related oxidative stress and are related to senescence of the cochlea and AHL in mice and humans [1, 2, 3, 4, 5]. Mitochondria are crucial for energy supply, maintaining cellular redox homeostasis, programmed cell death, and cell signaling regulation [6]. Translational precision by mitochondrial ribosome affects nuclear gene expression and cytoplasmic proteostasis, both of which are determinants of aging [7, 8, 9, 10]. The post‐transcription alteration of tRNA nucleotides occurs through numerous enzymatic reactions that are crucial for accurate and efficient decoding [11], mainly because they contribute to the binding of the tRNA codon [12, 13]. Several diseases, including mitochondrial diseases and type II diabetes, have been associated with alterations of mitochondrial tRNAs (mt‐tRNAs) [9, 10, 11, 14, 15, 16, 17, 18, 19, 20].

Mitochondrial tRNAs in mammals are modified by nuclear tRNA‐associated enzymes, including Cdk5 regulatory subunit‐associated protein 1 (CDK5RAP1), which induces 2‐methylthio (ms^2^) modifications in mammalian mt‐tRNAs [16, 21]. Incomplete deficiency in ms^2^ modification markedly weakens mitochondrial protein synthesis in stressful situations, leading to AHL and respiratory defects, as seen in *Cdk5rap1*‐knockout (KO) mice, which are predisposed to stress‐induced mitochondrial remodeling [22, 23]. In the inner ear of *Cdk5rap1*‐KO mice, deficiency in ms^2^ modification induces an initial decline in cochlear function owing to type II and IV spiral ligament (SLi) fibrocyte degeneration and stria vascularis (SV) senescence preceding spiral ganglion neuron (SGN) and hair cell (HC) loss (Fig. 1). The mitochondria of type II and IV SLi fibrocytes of *Cdk5rap1*‐KO mice were disrupted (i.e., mitochondria ballooning and damaged cristae) in the initial stages of AHL. These findings suggest that a reduction in the modification of mt‐tRNAs leads to SLi mitochondrial dysfunction and oxidative stress throughout the cochlea, thereby inducing cytotoxicity in fibrocytes and an early decrease in endolymphatic potential (EP) during the development of AHL.

**Fig. 1:**
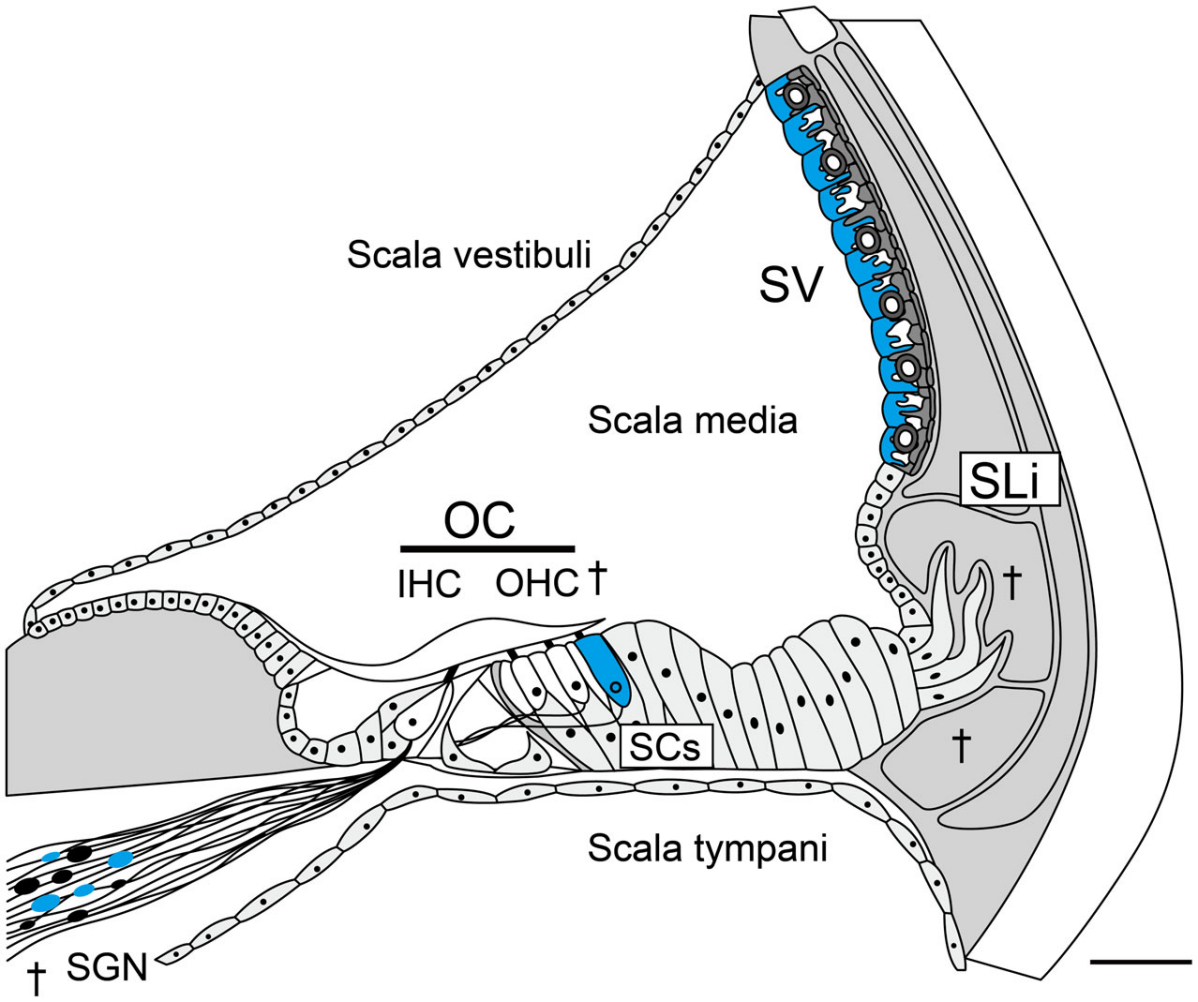
Cochlear senescence in *Cdk5rap1*‐KO mice. An illustration of a typical adult cochlea (cross‐section) shows the senescent cells in blue and degeneration (daggers) in *Cdk5rap1*‐KO mice compared with those in control (CNT) mice. The cochlea of an adult mammal is divided into three compartments: the tympani, media, and scala vestibuli. The organ of Corti is located in the scala media, which can be observed in this cross‐sectional image. Scale bar = 100 μm. OC, organ of Corti; SC, supporting cell.

The extracellular fluid present in the cochlear duct and ionic homeostasis of the endolymph is crucial for hearing and plays an important role in sound transduction [24]. The SV transports K^+^ ions and SLi fibrocytes travel from the organ of Corti via HCs back to the SV and SLi by transduction and then back into the scala media during this process [24, 25]. Chronic EP changes produced by ion content variations enforced by standing potentials may change the ionic composition of the endolymph [24]. Such putative ionic composition abnormalities of the local endolymph may lead to HC and secondary SGN corrosion, thereby causing detectable modifications in the dysfunctional lateral wall [24, 26]. Hearing is corrected by the ion‐transport system and mitonuclear protein homeostasis remodeling, which could restrain oxidative stress in young animals [6, 27].

Gradual dysfunction of the respiratory complexes in SLi fibrocytes occurs with age and affects the quality control of mitochondria, leading to SLi and SV cellular senescence and chronic EP reduction. The subsequent deformities in the ionic composition of the local endolymph may lead to HC deterioration and secondary SGN damage. Some studies have revealed that dysregulated tRNA modifications in humans precede aging [9, 10]. Thus, cochlear energy loss may cause AHL, possibly due to the reduction in post‐transcriptional mt‐tRNA modifications in humans.

However, previous studies did not further investigate outer HCs (OHCs), SGNs, marginal SV cells, or type I SLi fibrocytes even though damage or senescence was observed, which is crucial for histopathological assessment in AHL [24, 28, 29, 30] (Fig. 1). Therefore, the present study evaluated the cochlear mitochondria in the inner ears of *Cdk5rap1*‐KO mice that have reduced ms^2^ mt‐tRNA modifications.

## Methods

### Animals

*Cdk5rap1* and C57BL/6J mice (female, 64 weeks old) were donated by K. Tomizawa (Kumamoto University, Kumamoto, Japan). *Cdk5rap1* KO mice were bred by crossing transgenic mice with exons 5 and 6 of the *Cdk5rap1* floxed by *Lox*P sites with transgenic mice that express Cre recombinase under control of the CAG promoter. Backcrossing was performed with C57BL6/J mice for ≥ 7 generations to remove the Cre transgene and establish the genetic background. Control (CNT) and KO mice (4 weeks old) littermates were included in all experiments unless otherwise indicated. Detailed genotyping can be provided upon request. Groups of mice (comprising five mice in each group) were housed in a temperature‐controlled cage maintained at 25 °C and 50% humidity. All animals had unlimited access to commercial pellet food and water, and were randomly allotted to the experimental groups. All animal experiments were approved by the Committee on the Care and Use of Animals at Kumamoto University, Kumamoto, Japan (protocol number: H28‐053), and conducted in accordance with recognized standards of veterinary science for the Use of Humans and Animals in the Research of Neuroscience.

### Transmission electron microscopy

*Cdk5rap1‐*KO and CNT mice (male, five per group) were anesthetized via intraperitoneal injection of 4 mg·kg^−1^ of xylazine (Bayer, Shawnee, KS, USA) and 120 mg·kg^−1^ of ketamine‐HCl (Daiichi Sankyo, Tokyo, Japan) in NaCl (0.9%) at 4, 12, 20, or 48 weeks after birth. The mice were perfused with 2.5% glutaraldehyde (GLUT) and 2 mm CaCl_2_ formed in 0.1 m sodium cacodylate (C_2_H_6_AsNaO_2_) (pH 7.4) via the ascending aorta. The inner ears of mice were examined, and samples were maintained at 25 °C in a mixture of 2% paraformaldehyde and 2% GLUT in phosphate‐buffered saline (pH 7.4). Using 2% osmium tetroxide, postfixation was conducted on ice for 30 min. Consequently, the specimens were stained with 1.5% uranyl acetate for 1 h at 4 °C, desiccated in a 100% propylene oxide and ethanol gradient (50, 70, 90, and 100% in ascending order), inserted in Epon‐Araldite, and polymerized for 48 h at 60 °C. Samples were cut into 60–65‐nm sections with an ultramicrotome (UC7i; Leica, Munich, Germany) and stained with fresh Reynolds' lead citrate and 1.5% uranyl acetate at 25 °C. Transmission electron microscopy (TEM) (HT7700; Hitachi, Tokyo, Japan; *n* = 5 for each genotype) was used to analyze the samples. Twenty images of each sample were acquired at each magnification in the same plane for an accurate representation. The amount of damaged and normal mitochondria in each image was determined using the mitochondrion cristae score, which indicates structural abnormalities, in imagej software (National Institutes of Health, Bethesda, MD, USA) [31], and the proportion of wounded to normal mitochondria was calculated. In addition, we measured the dimensions of oblong mitochondria in each image (*n* = 5 for each genotype) by tracing the outer mitochondrial membrane of each mitochondrion using the freehand tool in imagej software (National Institutes of Health) [31].

### Statistical analyses

Eighty female *Cdk5rap1*‐KO mice and CNT littermates were selected for genotyping. The results were assessed using a two‐way ANOVA and a *post hoc* Tukey test. Statistical analyses were evaluated with graphpad prism 8.0.0 for Windows (GraphPad Software, San Diego, CA, USA). Data were corrected via Bonferroni analysis using the family‐wise error rate. The information validated the statistical test assumptions. The sample size and statistical power were estimated before and after the collection of data with ‘PS: Power and Sample Size Calculation’, Ver. 3.1.6 (Biostatistics Dept, Vanderbilt University, Nashville, TN, USA) [32]. Data are denoted as the mean ± standard error. *P* < 0.05 indicated statistical significance.

## Results

### Degenerating mitochondria were absent in HCs and SGNs of Cdk5rap1‐KO mice

**Fig. 2:**
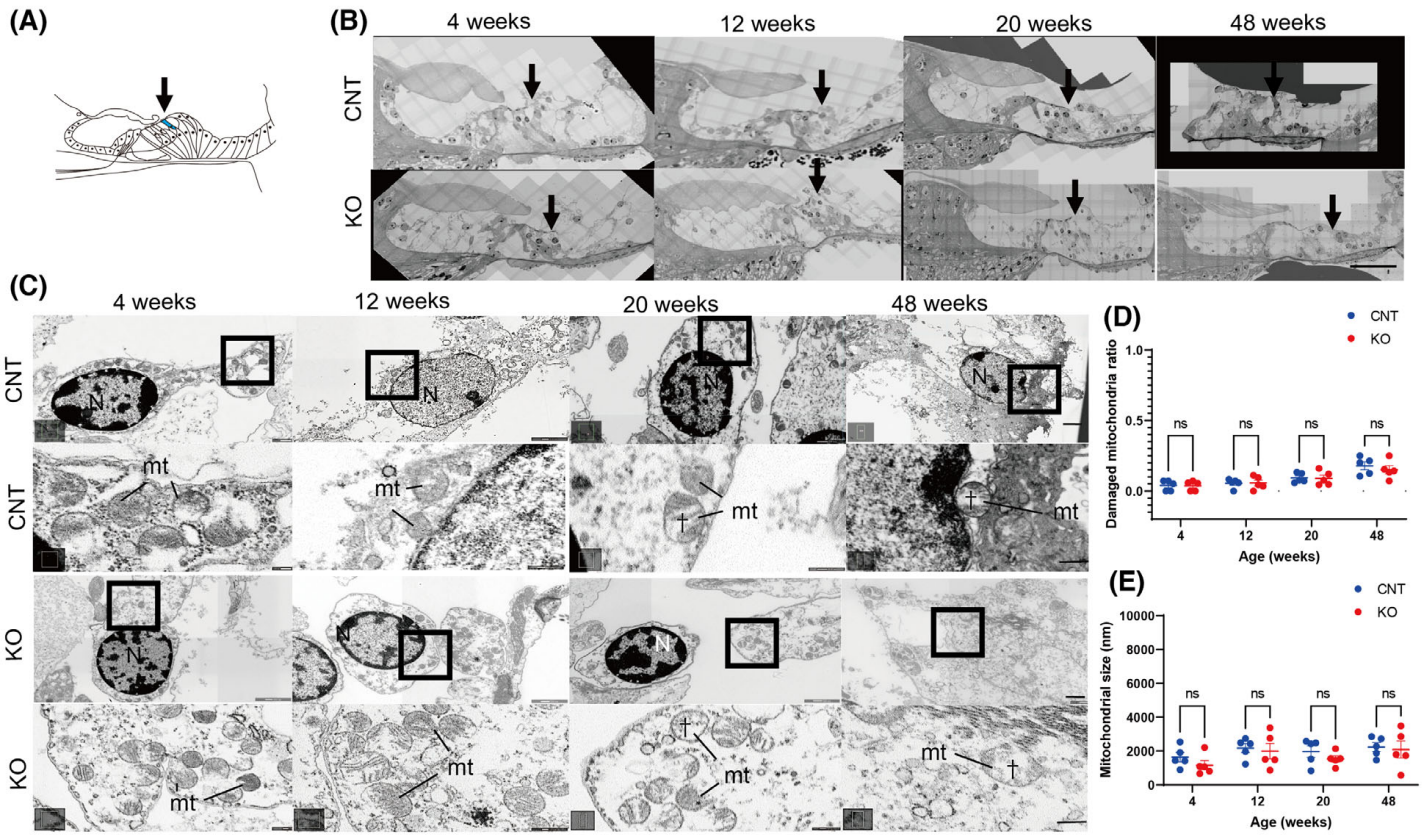
Normal mitochondria observed in the OHCs of *Cdk5rap1*‐KO mice. (A) A cross‐sectional image of the organ of Corti illustrates the distribution of senescent cells in blue in *Cdk5rap1*‐KO mice in contrast with control (CNT) mice. The arrow indicates the investigated cells. (B) TEM images of the OHCs from the cochlear middle turn. The arrow indicates the investigated cells. Scale bar = 100 μm. (C) TEM and magnified images of the third row of OHCs from the middle turn of the cochlea and mitochondrial cristae structure in *Cdk5rap1‐*KO or littermate CNT mice at various ages. Upper: CNT mice; lower: KO mice; mt: mitochondria; N: nuclei. Scale bar = 1 μm. Daggers denote mitochondrial cristae loss. In magnified images, scale bars = 100 nm. (D) Damaged mitochondria in OHCs of CNT and *Cdk5rap1*‐KO mice. (E) Mitochondrial size in OHCs in CNT and *Cdk5rap1*‐KO mice of various ages. Blue: CNT mice; red: *Cdk5rap1*‐KO mice; ns: not significant.

**Fig. 3:**
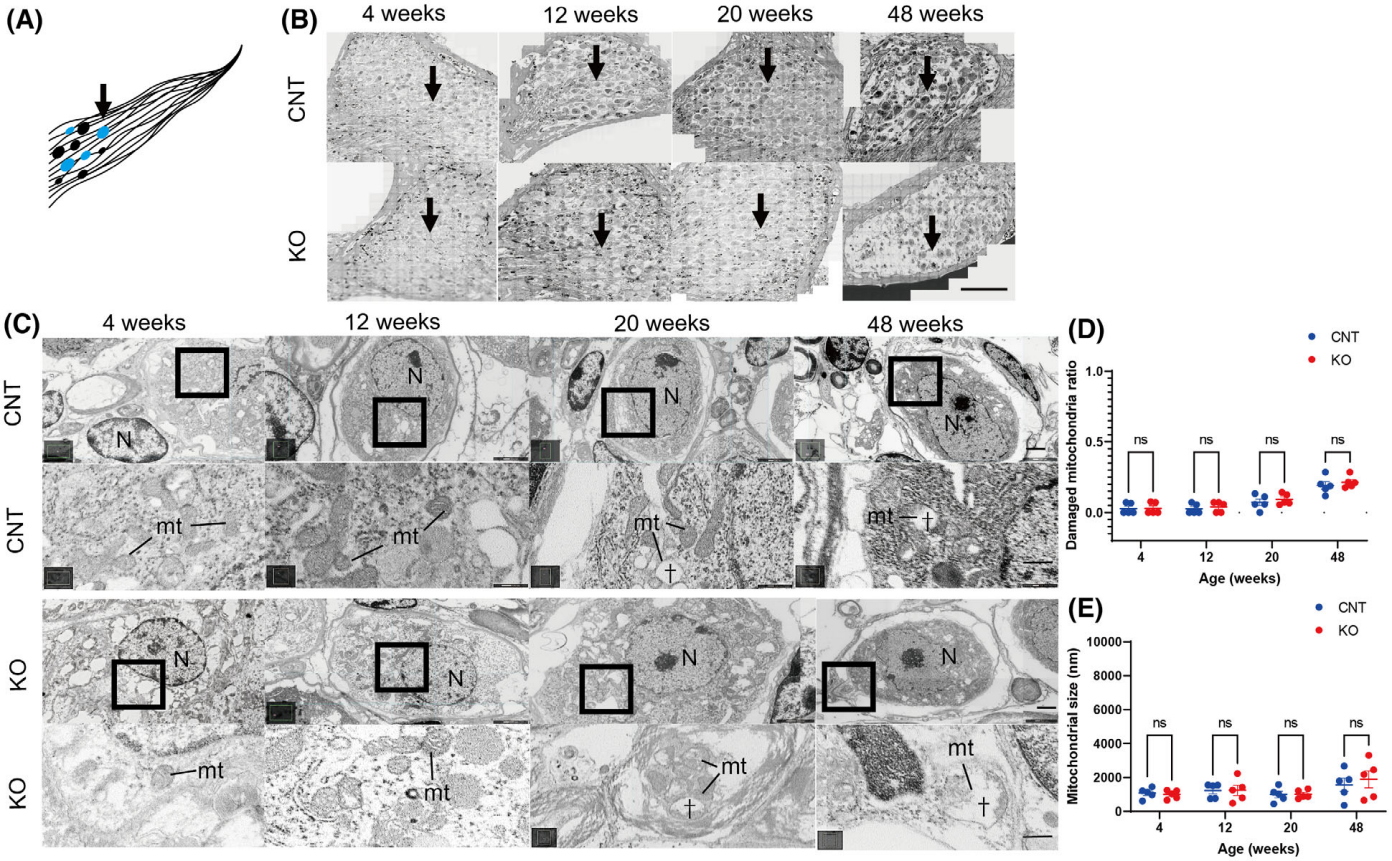
Normal mitochondria present in the SGNs of *Cdk5rap1*‐KO mice. (A) A cross‐sectional image of the SGN illustrates the distribution of the senescent cells in blue in *Cdk5rap1*‐KO mice compared with that in control (CNT) mice. Arrow indicates the investigated cells. (B) TEM images of SGNs from the cochlear middle turn. The arrow indicates the investigated cells. Scale bar = 100 μm. (C) TEM and magnified images of SGNs from the cochlear middle turn. The mitochondrial cristae structure of CNT or *Cdk5rap1‐*KO mice at various ages. Upper: CNT mice; lower: *Cdk5rap1*‐KO mice; mt: mitochondria; N: nuclei. Scale bar = 1 μm. Daggers indicate the absence of cristae of mitochondria. In magnified images, scale bars = 100 nm. (D) Damaged mitochondria in SGNs of CNT and *Cdk5rap1*‐KO mice. (E) Size of mitochondria in SGNs in CNT and *Cdk5rap1*‐KO mice of various ages. Blue: CNT mice; red: *Cdk5rap1*‐KO mice; ns: not significant.

Transmission electron microscopy revealed no differences in subcellular localization and its morphology, mitochondrial damage, and the sizes of HCs and SGNs according to age in *Cdk5rap1*‐KO compared with CNT mice (Figs 2A–E and 3A–E; Fig. S1A–E).

### Ballooning of mitochondria observed in the SV of Cdk5rap1‐KO mice

**Fig. 4:**
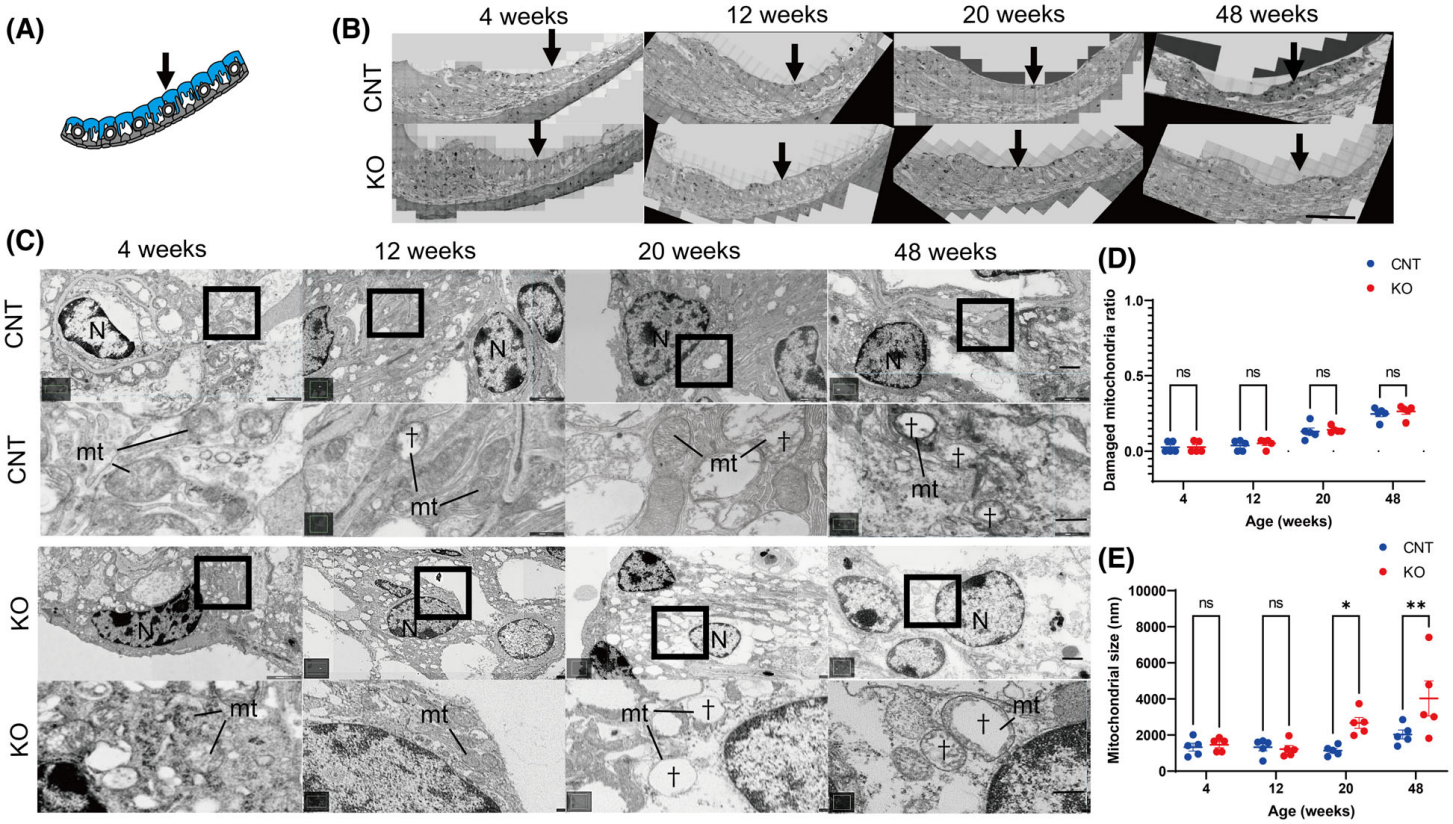
Ballooning of mitochondria observed in the SV of *Cdk5rap1*‐KO mice. (A) A cross‐sectional image of the SV illustrates the distribution of the senescent cells (blue) in *Cdk5rap1*‐KO mice versus that in control (CNT) mice. Arrow indicates the investigated cells. (B) TEM images of the SV from the cochlear middle turn. Arrow indicates the investigated cells. Scale bar = 100 μm. (C) TEM and magnified images of SV marginal cells from the cochlear middle turn. Age‐related changes in the mitochondrial cristae structure of *Cdk5rap1‐*KO or CNT mice. Upper: CNT mice; lower: KO; mt: mitochondria; N: nuclei. Scale bar = 1 μm. Daggers indicate mitochondrial cristae loss. In magnified images, scale bars = 100 nm. (D) Damaged mitochondria in the SVs of CNT and *Cdk5rap1*‐KO mice. (E) Size of mitochondria in the SVs of *Cdk5rap1*‐KO and CNT mice at various ages. Blue: CNT mice; red: *Cdk5rap1*‐KO; *P < 0.05; **P < 0.01; ns, nonsignificant.

Transmission electron microscopy revealed that compared with those in CNT mice, the mitochondria of SV marginal cells in *Cdk5rap1*‐KO mice did not lose the inner membrane cristae in the early and late stages (Fig. 4A–E). However, the mitochondria of *Cdk5rap1*‐KO mice were larger compared with those of CNT mice at 20 and 48 weeks of age (Fig. 4E; 20 weeks, *P* = 0.04; 48 weeks, *P* = 0.005; *F*[3, 32] = 3.401; *P* = 0.03). Degenerated mitochondria were not observed in basal and intermediate cells of the SV in *Cdk5rap1*‐KO mice (data not shown).

### Degenerated mitochondria in SLi fibrocytes of Cdk5rap1‐KO mice

**Fig. 5:**
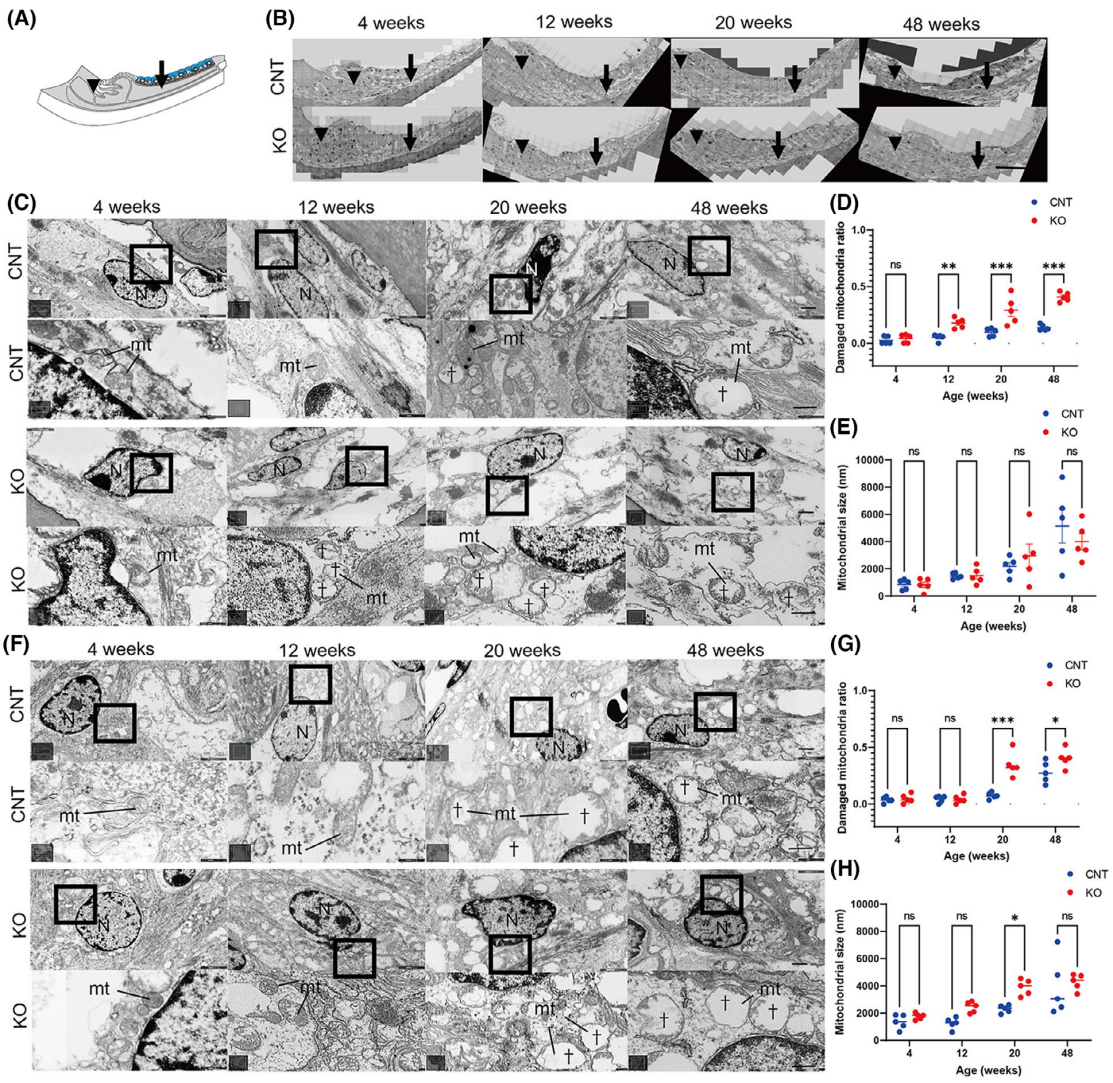
Disruptions of mitochondria are present in fibrocyte types I, II, and IV of the SLi in *Cdk5rap1*‐KO mice, with ballooning of mitochondria observed in type II and IV fibrocytes of the SLi in *Cdk5rap1*‐KO mice. (A) A cross‐sectional image of the cochlear lateral wall illustrates the distribution of senescent cells in blue in *Cdk5rap1*‐KO mice contrasted with that in control (CNT) mice. The arrow and arrowhead indicate the investigated cells. (B) TEM images of the SLi from the cochlear middle turn. Arrow and arrowhead indicate the investigated cells. Scale bar = 100 μm. (C) TEM and magnified images of type I fibrocytes of the SLi from the middle cochlear turn. In the cristae structure of the mitochondria, there were age‐related differences between *Cdk5rap1‐*KO and littermate CNT mice. Upper: CNT mice; lower: *Cdk5rap1‐*KO mice; mt: mitochondria; N: nuclei. Scale bar = 1 μm. Daggers denote mitochondrial cristae loss. In magnified images, scale bars = 100 nm. (D) Damaged mitochondria in type I fibrocyte of the SLi in CNT and *Cdk5rap1‐*KO mice. (E) Size of mitochondria in type I fibrocytes of the SLi in *Cdk5rap1‐*KO and CNT mice at various ages. (F) TEM and magnified images of fibrocytes (type II and IV) from the middle cochlear turn SLi. The cristae structure in the mitochondria of littermate CNT or *Cdk5rap1‐*KO mice at various ages. Upper: CNT mice; lower: *Cdk5rap1‐*KO; mt: mitochondria; N: nuclei. Scale bar = 1 μm. Daggers indicate mitochondrial cristae loss. In magnified images, scale bars = 100 nm. (G) Damaged mitochondria in fibrocytes (type II and IV) in *Cdk5rap1‐*KO and CNT mice SLis. (H) Size of mitochondrial in fibrocytes (type II and IV) of the SLi in *Cdk5rap1‐*KO and CNT mice at different ages. Blue: CNT mice; red: *Cdk5rap1‐*KO mice; *P < 0.05; **P < 0.01; ***P < 0.001; ns, nonsignificant.

Transmission electron microscopy showed that type I fibrocyte mitochondria in the SLi of *Cdk5rap1*‐KO mice had a loss of the cristae in the inner mitochondrial membrane (IM) starting at 12 weeks of age, which was earlier than that in CNT mice (Fig. 5A–E). At 12, 20, and 48 weeks of age, the mitochondria of *Cdk5rap1*‐KO mice were more noticeably impaired than those of CNT mice (Fig. 5D; 12 weeks, *P* = 0.004; 20 weeks, *P* < 0.001; 48 weeks, *P* < 0.001; *F*[3, 32] = 9.299, *P* < 0.001). However, the mitochondria of *Cdk5rap1*‐KO mice were not much bigger than those of CNT mice at any age (Fig. 5E). Regarding type II and IV SLi fibrocytes in relation to the findings of another study, the *Cdk5rap1*‐KO mice in our research began to lose the cristae in the IM at the age of 20 weeks (Fig. 5A,B,F). Furthermore, at 20 and 48 weeks of age, the mitochondria of *Cdk5rap1*‐KO mice were considerably more damaged than those of CNT mice (Fig. 5G; 20 weeks, *P* < 0.001; 48 weeks, *P* = 0.02; *F*[3, 32] = 10.07, *P* < 0.001). Furthermore, at 20 weeks of age, the mitochondria of *Cdk5rap1*‐KO mice were noticeably bigger than those of CNT mice (Fig. 5H; 20 weeks, *P* = 0.02; *F*[3, 32] = 18.77, *P* < 0.001).

## Discussion

We used TEM in *Cdk5rap1*‐KO mice to analyze the impact of insufficient ms^2^ modification of mt‐tRNAs, which results in poor mitochondrial protein translation in tissues in the cochlea during AHL. In 4‐week‐old *Cdk5rap1*‐KO mice, TEM revealed no differences in the mitochondrial structure and size compared with CNT mice. However, in older *Cdk5rap1*‐KO mice, TEM revealed changes in mitochondrial damage and size in the SV and SLi but not in the HCs and SGNs.

In a previous study, SGN and SV marginal cell senescence, SLi deterioration of fibrocytes (type II and IV), reduced production of Na^+^/K^+^ATPase α1 (fibrocytes, type II and IV) (Fig. 1), and a slight reduction in EPs was observed in younger *Cdk5rap1*‐KO mice. Conversely, reduced EPs and hearing loss were observed in older *Cdk5rap1*‐KO mice. The *Cdk5rap1*‐KO mice had SGN and HC loss as well as downregulation of Na^+^/K^+^‐ATPase α1 (fibrocytes, type II and IV) and Cx26 (type I fibrocytes) in the SLi at 20 weeks of age. Remarkably, compared with CNT mice, these alterations occurred sooner in *Cdk5rap1‐*KO mice (Fig. 1). In the aforementioned study, *Cdk5rap1*‐KO mice with SLi fibrocyte degeneration, low Na^+^/K^+^‐ATPase 1 expression, and SV senescence prior to the loss of HCs and SGNs showed an early decline in cochlear function. Therefore, we hypothesized that fibrocytes from the SLi counterbalance fewer EPs by moving ions through fibrocytes in the SLi and SV in younger *Cdk5rap1*‐KO mice.

Interestingly, in the present study, no discernible differences were found in mitochondrial damage and the sizes of HCs and SGNs at different ages between *Cdk5rap1*‐KO and CNT mice. AHL may be induced by HC and SGN damage according to the histopathology of the cochlea in age‐graded humans and mice [33]. HC damage is exacerbated by age, whereas SGN damage is constant with the disintegration that occurs after the loss of HCs. Although mitochondrial damage is considered the cause of age‐related degeneration of HCs and SGNs, our study suggests that mitochondrial damage in HCs and SGNs does not lead to the onset of AHL. We speculated that the reason for these results was tissue damage due to chronic EP drop, as described below.

In addition to the loss of HCs and their innervation via SGNs, cochlear histopathology has also reported widespread degeneration of fibrocytes from SLi [2, 24] and SV degeneration. Our study showed that although mitochondrial damage in type I SLi was observed in *Cdk5rap1*‐KO mice at 12 weeks of age, the ballooning was not significant compared with CNT. Moreover, at the same stage, mitochondria in type II and IV SLi were disrupted (i.e., mitochondria ballooning and cristae loss). As a result, HC and SGN damage was preceded by the cell damage identified in the SLis of *Cdk5rap1‐*KO mice as well as EP reduction at a younger age, indicating that the fibrocyte pathology resulting in the degeneration of sensory cells may be the cause of early AHL since such tendencies were seen at older ages in CNT mice. Previous studies have reported that the administration of a permanent complex II blocker or a mitochondrial toxin, such as 3‐nitropropionic acid, caused inflammatory responses in the lateral wall following acute mitochondrial dysfunction [34, 35]. Moreover, these previous studies have also described lateral wall histopathology and mitochondrial dysmorphology.

Regarding SV, in our previous study, senescence markers were expressed in the marginal SV cells; however, despite its significance in the pathophysiology of AHL, we did not observe any modifications in the thickness of the SV with aging [24, 36]. TEM analysis showed ballooned, although undamaged, mitochondria in the marginal SV cells in 20‐week‐old *Cdk5rap1‐*KO mice. We speculate that this was caused by SLi compensation, as the SV functions as a barrier between the lateral wall and the endolymphatic area.

In *Cdk5rap1‐*KO mice, dysfunction of the mitochondria is counterbalanced by mitonuclear protein homeostasis [22] through the initiation of the unfolded protein response of mitochondria that applies a defensive effect [6, 22]. The amount of mitochondrial damage probably exceeds the ability of mitophagy to maintain the mitochondrial network, which results in mitochondrial malfunction [22, 37]. Additionally, elevated complex I deficiencies were linked to a slight increase in oxidative stress, which results in the unfolded protein response and cytotoxicity [37]. Our research corroborates the idea that, depending on its severity, mitochondrial malfunction under sedentary conditions may not instantly result in a pathogenic phenotype. These findings imply that the lack of mt‐tRNA alterations generates oxidative stress throughout the cochlea and failure of mitochondria in SV and SLi, which triggers fibrocyte cytotoxicity and reduces EPs early on in the advancement of AHL.

Based on our research, we hypothesized that AHL in *Cdk5rap1*‐KO mice is caused by the depletion of the ion‐transport system in and around the SV (i.e., SLi fibrocytes) affected by reduced mitochondrial activity as a result of the lack of ms^2^ modifications in mt‐tRNAs. By altering the ion‐transport system and homeostasis of mitonuclear proteins, hearing is compensated, which may prevent oxidative stress in young animals [6, 27]. As people age, the increasing disruption of the respiratory complexes affects the quality control of the mitochondria in SLi fibrocytes, leading to senescence of the SLi and SV cells and a persistent drop in EPs. The loss of secondary SGNs and subsequent HC degeneration may be facilitated by the ensuing anomalies in the local endolymphatic ion composition. Previous research has demonstrated that dysregulated tRNA changes occur before human aging [9, 10]. As a result, reduced post‐transcriptional alterations in mt‐tRNAs in humans may be the source of AHL.

The C57BL/6 mouse model might not accurately depict AHL, even though the pathology and etiology of hearing loss are identical to those of AHL. This is a major limitation of our *in vivo* investigation. Due to a *Cdh23* mutation, C57BL/6 mice are more susceptible to AHL [38]. Ideally, we would have used strains such as BALB/c or CBA that were backcrossed with a strain without any type of hearing issues. Moreover, TEM artifacts were observed in this study and vacuolization from inadequate fixation can be seen in microstructural TEM images as artifacts frequently. Therefore, adequate settings and conditions with sufficient fixation are necessary for future studies.

The findings of previous and current studies suggest that ms^2^ modifications of mt‐tRNAs may cause oxidative stress and damage to the mitochondria in the SLi and accelerate aging, thereby causing AHL. In our study, the mitochondrial findings in HCs and SGNs were normal, despite senescence. Mitochondrial findings in the SV revealed only ballooning, and those in the SLi type I, II, and IV fibrocytes indicated damage and ballooning. Generally, the presence of mitochondria has been reported in inner hair cells (IHCs), OHCs, SGNs, SV, and SLi in the cochlea. Mitochondria are located at the top and around the nucleus in IHCs, whereas mitochondria are located at the top and along the outer wall of the OHCs. Mitochondria are located in the fine membranous processes of the marginal cells in the SV [39, 40].

Mitochondria play a crucial role in providing energy for the specialized sensory HCs that detect sound and enable hearing. In addition to their energy‐producing role, mitochondria in the inner ear also play a role in regulating the calcium levels in HCs, which is important for their function. Mitochondria are also involved in the process of apoptosis, which can be triggered by certain types of damage or stress to the HCs. Overall, the physiological function of mitochondria in the inner ear is critical for the proper function of the sensory HCs and, thereby, for hearing [39]. Our study suggested that mitochondria in the SLi were also crucial for hearing.

We demonstrated that a lack of CDK5RAP1, which catalyzes modifications of mt‐tRNAs (ms^2^), results in mitochondrial malfunction. Cell senescence and AHL are caused by mitochondrial malfunction. Our findings imply that defective mitochondria may contribute to the advancement of AHL. Our analysis provides valuable insights regarding the underlying mechanisms of AHL and the relationship between aberrant tRNA modification‐induced hearing loss and mitochondrial dysfunction. Future treatment approaches aimed at reducing the consequences of AHL could be guided by elucidating the processes causing AHL.

## Conflict of interest

The authors declare no conflict of interest.

### Peer Review

The peer review history for this article is available at https://www.webofscience.com/api/gateway/wos/peer-review/10.1002/2211-5463.13655.

## Author contributions

TM conceived and designed the project. TM and TK acquired the data. TM analyzed and interpreted the data and wrote the paper. F‐YW and KT supervised the project. All authors read and approved the final manuscript.

## Supporting information


**Fig. S1.** Normal mitochondria are present in the inner hair cells of *Cdk5rap1‐*KO mice. (A) A cross‐sectional image of the organ of Corti illustrates the distribution of senescent cells (blue) in *Cdk5rap*‐knockout (KO) mice compared with that in control (CNT) mice. Arrow indicates the investigated cells. (B) TEM images of the inner hair cells (IHCs) from the cochlear middle turn. Arrow indicates the investigated cells. Scale bar = 100 μm. (C) Transmission electron microscopy (TEM) and magnified images of inner hair cells (IHCs) from the cochlear middle turn. The structure of cristae in the mitochondria of *Cdk5rap1‐*KO or littermate CNT mice at different ages. Upper: CNT mice; lower: *Cdk5rap1‐*KO mice; mt: mitochondria; N: nuclei. Scale bar = 1 μm. Daggers indicate the loss of mitochondrial cristae. In magnified images, scale bars = 100 nm. (D) Ratio of damaged mitochondria in IHCs in *Cdk5rap1‐*KO and CNT mice. (E) Mitochondrial size of IHCs in *Cdk5rap1‐*KO and CNT mice of different ages. Red: CNT mice; blue: *Cdk5rap1‐*KO; ns: not significant.

## Data Availability

The data that support the findings of this study are available from the corresponding author, TM, upon reasonable request.
