# Supplementary material for: Mitochondrial alterations in the cochlea of Cdk5rap1‐knockout mice with age‐related hearing loss
Source: FEBS Open Bio. 2023 Jun 6;13(7):1365–74. doi: 10.1002/2211-5463.13655 (PMC10315731; doi:10.1002/2211-5463.13655)
Supplement: Supplementary file 1 — Fig. S1. Normal mitochondria are present in the inner hair cells of Cdk5rap1‐KO mice. (A) A cross‐sectional image of the organ of Corti illustrates the distribution of senescent cells (blue) in Cdk5rap‐knockout (KO) mice compared with that in control (CNT) mice. Arrow indicates the investigated cells. (B) TEM images of the inner hair cells (IHCs) from the cochlear middle turn. Arrow indicates the investigated cells. Scale bar = 100 μm. (C) Transmission electron microscopy (TEM) and magnified images of inner hair cells (IHCs) from the cochlear middle turn. The structure of cristae in the mitochondria of Cdk5rap1‐KO or littermate CNT mice at different ages. Upper: CNT mice; lower: Cdk5rap1‐KO mice; mt: mitochondria; N: nuclei. Scale bar = 1 μm. Daggers indicate the loss of mitochondrial cristae. In magnified images, scale bars = 100 nm. (D) Ratio of damaged mitochondria in IHCs in Cdk5rap1‐KO and CNT mice. (E) Mitochondrial size of IHCs in Cdk5rap1‐KO and CNT mice of different ages. Red: CNT mice; blue: Cdk5rap1‐KO; ns: not significant. [file FEB4-13-1365-s001.docx]

**Supplementary Material**


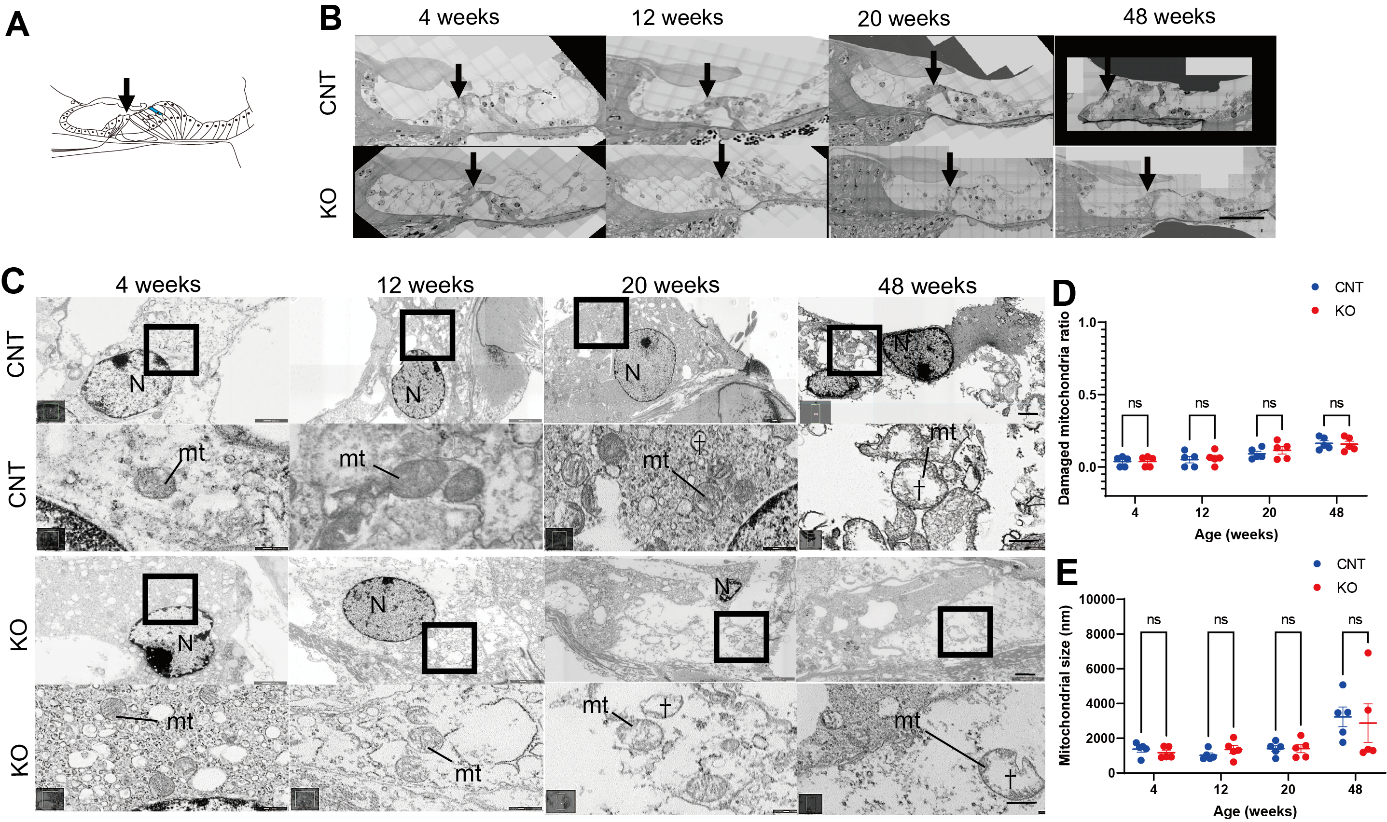
 **Supplementary Figure 1. Normal mitochondria are present in the inner hair cells of *Cdk5rap1-*KO mice.** (A) A cross-sectional image of the organ of Corti illustrates the distribution of senescent cells (blue) in *Cdk5rap*-knockout (KO) mice compared with that in control (CNT) mice. Arrow indicates the investigated cells. (B) TEM images of the inner hair cells (IHCs) from the cochlear middle turn. Arrow indicates the investigated cells. Scale bar = 100 µm. (C) Transmission electron microscopy (TEM) and magnified images of inner hair cells (IHCs) from the cochlear middle turn. The structure of cristae in the mitochondria of *Cdk5rap1-*KO or littermate CNT mice at different ages. Upper: CNT mice; lower: *Cdk5rap1-*KO mice; mt: mitochondria; N: nuclei. Scale bar = 1 µm. Daggers indicate the loss of mitochondrial cristae. In magnified images, scale bars = 100 nm. (D) Ratio of damaged mitochondria in IHCs in *Cdk5rap1-*KO and CNT mice. (E) Mitochondrial size of IHCs in *Cdk5rap1-*KO and CNT mice of different ages. Red: CNT mice; blue: *Cdk5rap1-*KO; ns: not significant.
